# Assessing the Potential for Genome-Assisted Breeding in Red Perilla Using Quantitative Trait Locus Analysis and Genomic Prediction

**DOI:** 10.3390/genes14122137

**Published:** 2023-11-27

**Authors:** Sei Kinoshita, Kengo Sakurai, Kosuke Hamazaki, Takahiro Tsusaka, Miki Sakurai, Terue Kurosawa, Youichi Aoki, Kenta Shirasawa, Sachiko Isobe, Hiroyoshi Iwata

**Affiliations:** 1 Graduate School of Agricultural and Life Sciences, The University of Tokyo, Bunkyo, Tokyo 113-8657, Japan; kinoshita@ut-biomet.org (S.K.); sakurai@ut-biomet.org (K.S.); 2 RIKEN Center for Advanced Intelligence Project, Kashiwa, Chiba 227-0871, Japan; kosuke.hamazaki@riken.jp; 3 TSUMURA & CO., Ami, Ibaraki 300-1155, Japan; tsusaka_takahiro@mail.tsumura.co.jp (T.T.); sakurai_miki@mail.tsumura.co.jp (M.S.); kurosawa_terue@mail.tsumura.co.jp (T.K.); aoki_youichi@mail.tsumura.co.jp (Y.A.); 4 Kazusa DNA Research Institute, Kisarazu, Chiba 292-0818, Japan; shirasaw@kazusa.or.jp (K.S.); sisobe@kazusa.or.jp (S.I.)

**Keywords:** breeding, genomic selection, medicinal plants, quantitative trait locus analysis, red perilla

## Abstract

Red perilla is an important medicinal plant used in Kampo medicine. The development of elite varieties of this species is urgently required. Medicinal compounds are generally considered target traits in medicinal plant breeding; however, selection based on compound phenotypes (i.e., conventional selection) is expensive and time consuming. Here, we propose genomic selection (GS) and marker-assisted selection (MAS), which use marker information for selection, as suitable selection methods for medicinal plants, and we evaluate the effectiveness of these methods in perilla breeding. Three breeding populations generated from crosses between one red and three green perilla genotypes were used to elucidate the genetic mechanisms underlying the production of major medicinal compounds using quantitative trait locus analysis and evaluating the accuracy of genomic prediction (GP). We found that GP had a sufficiently high accuracy for all traits, confirming that GS is an effective method for perilla breeding. Moreover, the three populations showed varying degrees of segregation, suggesting that using these populations in breeding may simultaneously enhance multiple target traits. This study contributes to research on the genetic mechanisms of the major medicinal compounds of red perilla, as well as the breeding efficiency of this medicinal plant.

## 1. Introduction

*Perilla frutescens* (perilla), an annual herb belonging to the Labiatae family native to China, is widely cultivated in China, Japan, Korea, and other Asian countries [1]. Perilla has long been used as an ingredient in food and traditional (Kampo) medicine. Perilla can be categorized into two types based on morphological differences in its leaves: red perilla (‘aka-jiso’), which has dark red or purple leaves and stems due to the presence of anthocyanin, and green perilla (‘ao-jiso’), which only contains a small amount of anthocyanin and has green leaves [2,3]. Red perilla is used as an ingredient in Kampo medicines, often to ease the stomach, cold, and anxiety, and is blended into several formulations, such as Hangekobokuto and Kososan. The main medicinal compounds in perilla are perillaldehyde (PA), rosmarinic acid (RA), and anthocyanins (ANT) [4]. PA is an odorant with antidepressant, anticancer, and antibacterial properties, whereas RA is a phenol with anti-inflammatory and antioxidant properties and is more abundant in green perilla, and ANT is a red pigment with antioxidant properties [2,3,5]. Owing to its benefits, red perilla has recently attracted attention as an ingredient in Kampo medicine, both in Asian countries and globally [6]. Therefore, the development of varieties with stable medicinal compounds is necessary to meet the increasing demand for red perilla. Although extensive research has been conducted on the bioactivity, metabolic pathways, and efficacy of red perilla medicinal compounds, no studies have investigated the enhancement of these medicinal compounds via selective breeding.

The quality of perilla plants used in Kampo medicine is determined by the concentrations of the major medicinal compounds: PA, RA, and ANT. Therefore, these compounds are considered the target traits for breeding red perilla as a medicinal plant. Conventional phenotype-selection-based breeding is not an efficient breeding method for medicinal plants because it requires extensive quantification of medicinal compounds in every individual of each generation, which is both expensive and time-consuming. Compound analysis involves plant sampling, drying, sample grinding, reagent preparation, compound extraction, quantification using ultra-high-performance liquid chromatography (UPLC), and data organization. These elaborate protocols take up to 1 month for even experienced individuals, therefore halting other experiments. In addition, there are economic costs involved in field management and labor. Genomic selection (GS) [7], an alternative approach in which genomic prediction (GP), is used to predict the phenotype from genome-wide marker information, which is suitable for improving medicinal traits. In GS, once a GP model is built, phenotypes can be predicted based solely on genomic information without the need to quantify medicinal compounds for every generation. If a target trait is controlled by a small number of quantitative trait loci (QTLs), marker-assisted selection (MAS), a selection method using markers that are strongly linked to the QTLs that control the target trait, can also be used for selection using only the genotypes of the linked markers. Genomic information can be obtained during the early growth stages of a plant, eliminating the need for field cultivation and accelerating the breeding cycle. With the development of next-generation sequencing (NGS) technology, obtaining genomic information has become easier and more economical. Therefore, GS and MAS are better suited than phenotypic selection for breeding red perilla since they minimize field cultivation and compound quantification.

GS was first proposed by Meuwissen et al. in 2001 and has since been successfully implemented in dairy cattle breeding [7,8]. In plant breeding, GS has been applied in major crops such as maize and rice, as well as other plants [9,10,11]. Most previous studies using GS have focused on yield or weight as target traits; however, some studies have focused on nutrients and other active ingredients, such as vitamin E in sweet corn, carotenoids in maize, or various metabolites in oats [12,13,14]. GS, focusing on active ingredients, is a crucial method in plant breeding. However, no studies have investigated GS in medicinal plants using medicinal compounds as target traits. Furthermore, in breeding to improve medicinal compounds, it is essential to target multiple compounds simultaneously. However, few such studies have been conducted. When several traits are improved simultaneously, it is important to consider both the correlation among the traits and the correlation between medicinal compounds and other important agronomic traits, such as yield and flowering. Therefore, it is insufficient to focus only on the phenotypic correlations among these traits. Genetic architecture and correlations estimated by genetic relationship matrices should also be utilized to assess whether each trait could be improved independently.

As few elite cultivars of red perilla exist for medicinal use, it is difficult to improve multiple medicinal compounds using only one population generated through a single cross combination. In this study, we used three populations generated by crossing red and green perilla to simultaneously enhance multiple medicinal compounds. When multiple populations with different genetic backgrounds are used, genetic correlations among traits, heritability, and accuracy of GPs are likely to differ among populations. Therefore, it is necessary to evaluate the extent of variation in these values across populations and whether the results differ from those obtained when predictions are made using all three populations together.

The present study had two main objectives: to evaluate the possibility of improving major medicinal compounds and other agronomic traits simultaneously in red perilla through genomic breeding and to identify the genetic characteristics of each population arising from differences in the effects of alleles derived from different parents and different levels of segregation in consequent generations. To achieve these objectives, we determined the genetic correlations among the traits mentioned above and conducted QTL and GP analyses using three populations with different genetic backgrounds. Based on the results of these analyses, the genetic architecture of each trait and the potential for breeding using GS and MAS are discussed. The results of this study provide valuable insights into the genetic mechanisms underlying the production of the major medicinal compounds of red perilla and will contribute substantially to red perilla breeding.

## 2. Materials and Methods

### 2.1. Plant Materials

The plant materials used in this study were the breeding populations of the F_3_ and F_4_ generations of two-way crosses between red and green perilla. The breeding population for each generation consisted of three populations. The cross-parents of these populations were ‘SekihoS8’, st27, st40, and st44. ‘SekihoS8’ is a representative variety of red perilla (*P. frutescens* var. *crispa f. purpurea*) owned by TSUMURA & CO. (Ami, Ibaraki, Japan), while st27, st40, and st44 were green perilla varieties (*P. frutescens Britton* var. *crispa Decne*) owned by the National Agricultural and Food Research Organization. The cross combinations were ‘SekihoS8’ × st27 (S827), ‘SekihoS8’ × st40 (S840), and ‘SekihoS8’ × st44 (S844), hereafter referred to as S827, S840, and S844, respectively. The plants were grown in the Ibaraki field, TSUMURA & CO, in 2021 and 2022.

**Figure 1:**
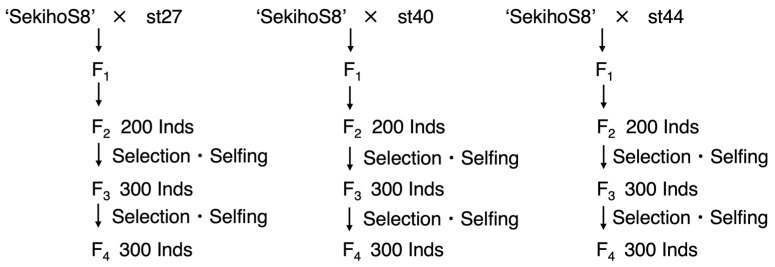
Selection and breeding scheme for the breeding populations used as plant parent materials in this study.

The selection and breeding schemes used to derive the breeding populations are shown in Figure 1. The breeding scheme was advanced through repeated self-pollination after an initial cross with the parents mentioned above. In the F_2_ generation, 200 lines were grown from each of the three crosses. From these, 100 lines from each population were selected for self-pollination, and three seeds per line were collected and cultivated for the F_3_ generation. To maintain the genetic diversity of the population, in the F_3_ generation, 50 lines were selected from those grown in the field, and 50 lines were selected from those not grown in the field for each population. Three individuals were cultivated in the next generation, F_4_, from the seeds collected from each line.

### 2.2. Phenotype Data

In the F_3_ population, perillaldehyde (PA), rosmarinic acid (RA), and anthocyanin (ANT) content were measured in plants grown in the experimental field. In the F_4_ population, PA, RA, yield, and flowering date were measured for plants grown in the experimental field.

PA and RA were quantified as described previously [15,16]. Briefly, to determine PA content, 0.1 g of each plant was weighed, placed in 25 mL of methanol, shaken, and centrifuged. The supernatant was separated and analyzed using UPLC at a wavelength of 234 nm using a UV detector. To determine the RA content, 0.05 g of each plant was weighed, placed in a 25 mL methanol/water (3:1) solution, shaken, and centrifuged, after which the supernatant was separated and analyzed by UPLC at a wavelength of 330 nm using a UV detector. The ANT content was measured thrice on 14 July, 29 July, and 12 August 2021. For each measurement, the ANT content was measured using three fully expanded leaves per plant with an anthocyanin content meter, and the average was recorded. The yield was measured thrice on 19 July, 29 July, and 8 August 2022, and all three measurements were added for subsequent analysis. For each yield measurement, the plants were harvested at a height of 45 cm using a machine, and the weight of the harvested plants was measured. The survey on flowering dates was conducted from 31 August 2022 to 30 September 2022. The number of days from 25 April, the date of sowing in the cell trays, to the flowering date was used for data analysis.

The phenotypic data for the four cross-parents collected in 2022 are shown in Table S1. SekihoS8 has a red leaf color, which maintains the content of ANT, whereas st27, st40, and st44 are superior in PA, RA, and YI to SekihoS8.

### 2.3. Genotype Data

Genomic DNA was extracted from all the individuals in the F_3_ and F_4_ generations, as well as from the four parental lines, ‘SekihoS8’, st27, st40, and st44. Double-digest restriction-associated DNA sequencing [17] was performed using a DNBSeq-G400RS (MGI Tech Co., Ltd. Shenzhen, China) to examine the genome-wide single nucleotide polymorphisms (SNPs). The 100 paired-end reads obtained were mapped to the reference sequence Hoko-3 (*P. frutescens cv.*), as published by Tamura et al. [18] using Bowtie2 [19]. Variant calling (max-missing 0.9) was conducted, and low-quality SNPs were filtered out using the VCFtools version 0.1.16 [20], after which 2282 SNPs were identified. SNPs with minor allele frequencies (MAF < 0.025) were excluded, and missing data were included using Beagle version 5.1 [21], after which they were filtered again using MAF < 0.025. Finally, 2063 SNPs were identified. These SNPs were filtered with MAF < 0.01 for both each population and the total three populations. In the F_3_ generation, 1964 SNPs remained in the entire population, including 727, 1432, and 579 each in S827, S840, and S844, respectively. In the F_4_ generation, 1964 SNPs remained in the entire population, including 862, 1432, and 579 in S827, S840, and S844, respectively. For further analysis, the score for SNPs of the same genotype as ‘SekihoS8’ was set to 0, 1 for heterozygous SNPs, and 2 for homozygous SNPs in the other parent.

### 2.4. Linkage Map Construction and QTL Analysis

The perilla used in this study was an allotetraploid (2n = 4x = 40) species with a genome size of about 1.24 Gb [18]. The F_3_ generation was used to create a linkage map, and the 1964 SNPs described in the previous subsection were used as marker genotypes. The order of the markers was the same as that described in Section 2.3. Based on the expected ratio of 3:2:3 for the marker classes at each locus, the Maximum Likelihood Method was used to estimate the recombination rates between markers for each population. Markers with recombination rates greater than 0.499 were excluded and the remaining markers were transformed into map distances using the Kosambi function [22]. Subsequently, to obtain a common linkage map among the populations, smoothing was performed among the three populations using the ‘loess’ function in the ‘stats’ package version 4.1.2 in R. A common linkage map is shown in Figure S1.

Based on the common linkage map described above, QTL analysis was performed for each population using the ‘cim’ function in the ‘qtl’ package version 1.60 in R [23]. The window size was set to 10 cM, and the covariate markers were set to five. Logarithm of odds (LOD) scores were determined using 10,000 permutations. The targeted traits in the QTL analysis were PA, RA, and ANT in the F_3_ generation and PA, RA, yield, and flowering date in the F_4_ generation.

### 2.5. Genomic Heritability and GP Model

Genomic heritability was estimated for all the traits described in Section 2.2, and single- and multi-trait GP models were constructed for each generation. GPs were generated for each population and the three combined populations.

To evaluate the GP accuracy, a 10-fold cross validation was performed and repeated 10 times. For each cross validation, the Pearson correlation between the observed and predicted values was calculated, and the average of these correlations was used as the prediction accuracy.

#### 2.5.1. Single Trait GP Model

The GBLUP [24] and BayesB [7] models were used for single-trait GP. The GBLUP model can be expressed as:

**y** = **Xβ** + **Zu** + **ε**, (1)

where n is the number of individuals, g is the number of genotypes, **y** is an n × 1 vector representing phenotypic values of a target trait, **Xβ** is an n × 1 vector corresponding to fixed effects, **Zu** is an n × 1 vector corresponding the term of random effects. In the prediction model for each population, **Xβ** represents an intercept, whereas in the model for all three populations, **X** represents an n × 3 design matrix, **β** is a 3 × 1 vector, and **Xβ** is a term correcting for the average effect of each population. The random effect **u** is a g × 1 vector corresponding to the genotypic values that follow a multivariate normal distribution.

**u** ~ MVN(**0**, **G**σ^2^_u_), (2)

where **G** is a g × g additive genomic relationship matrix, and σ^2^_u_ is a genetic variance. In this study, the additive genomic relationship matrix **G** was computed based on the marker genotype data using the ‘calcGRM’ function in the ‘RAINBOWR’ package version 0.1.29 in R [25]. The additive relationship matrix G was computed as a linear kernel of the marker genotype scaled by the allele frequency divided by the normalization constant, as described previously [26]. **ε** is an n × 1 error vector that follows a multivariate normal distribution;

**ε** ~ MVN(**0**, **I_n_**σ^2^_e_), (3)

where **I_n_** is an n × n identity matrix, and σ^2^_e_ is an error variance. We estimated the genetic variance σ^2^_u_ and the error variance σ^2^_e_ using the ‘EMM.cpp’ function in the ‘RAINBOWR’ package version 0.1.29 in R [24]. Based on the estimated genetic and error variances, the genomic heritability for each trait was calculated as h^2^ = σ^2^_u_/(σ^2^_u_ + σ^2^_e_).

The BayesB model can be written as:

**y** = **Xβ** + **Wa** + **ε**, (4)

where m is the number of markers, **W** is an n × m matrix of the marker genotype, and **a** is an m × 1 vector representing marker effects. The model was implemented using the ‘BGLR’ function in the ‘BGLR’ package version 1.1.0 in R [27].

#### 2.5.2. Multi Trait GP Model

GBLUP [28] and BayesCπ [29] models were used for multi-trait GP. The GBLUP model is similar to Equation (1), but with the vector of phenotypic values **y** extended to an nt × 1 vector through the vertical alignment of the phenotypic values of ‘t’ traits. Additionally, the random effect **u** now follows a multivariate normal distribution **u** ~ MVN(**0**, **K** ⊗ **G**), where **K** is a t × t genetic variance–covariance matrix between the t traits, and **G** is the g × g additive genetic matrix. The non-diagonal elements of **K** represent covariances between traits. The error **ε** follows a multivariate normal distribution **ε** ~ MVN(**0**, **R** ⊗ **I_n_**), where **R** is the t × t residual variance–covariance matrix of the traits, and **I_n_** is the n × n identity matrix. Here, **A** ⊗ **B** represents the Kronecker product between the two matrices, A and B. The BayesCπ model is similar to Equation (4). The model was also extended to multi-trait GP by considering the covariances between the different ‘t’ traits at each marker. Furthermore, in BayesB for the single-trait model, the prior distribution of each marker effect is a scaled-t distribution, whereas, in the BayesCπ for the multi-trait model, a t × 1 vector of each marker effect for t traits follows a normal prior distribution. The multi-trait GP models were implemented using the ‘Multitrait’ function in the ‘BGLR’ package version 1.1.0 in R [27].

## 3. Results

### 3.1. QTL Analysis

**Figure 2:**
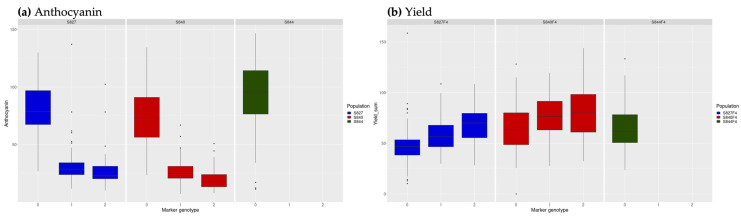
Genotypes at the detected quantitative trait loci (QTLs) identical to anthocyanin and yield. Genotype scores 0, 1, and 2 represent homozygous for ‘SekihoS8’, heterozygous, and homozygous for the other cross parents, respectively. Blue: S827; Red: S840; Green: S844. (**a**) The genotypes at the detected QTL on chr8 for anthocyanin (1st measurement); (**b**) genotypes at the detected QTL on chr8 for yield.

We performed QTL analysis in each population and each generation. In the F_3_ generation, the three target traits (PA, RA, and ANT) were investigated. In the F_4_ generation, PA, RA, yield, and flowering date were investigated. Following composite interval mapping, 136 QTLs in the F_3_ generation and 268 QTLs in the F_4_ generation were detected above the threshold LOD score. The number of QTLs consistent across multiple populations or two generations was 48, 0, 2, 2, and 11 for PA, RA (Figure S2), ANT (Figure S3), yield (Figure S4), and flowering date (Figure S5), respectively. Among these QTLs, two each for ANT and yield were detected at identical locations. These two QTLs may represent a single pleiotropic QTL. As shown in Figure 2a, when the genotype at the detected QTL was homozygous for ‘SekihoS8’ (red perilla), the phenotypic value of ANT content was also higher. In S844, no QTLs were detected, and all individuals were homozygous for ‘SekihoS8’ at the corresponding markers. However, when the genotype at the QTLs detected for yield was homozygous for ‘SekihoS8’, the phenotypic value of yield was lower than that of the other genotypes (Figure 2b).

Eleven QTLs for flowering date were detected across multiple populations, of which 1 QTL was common to S827 and S844, and 10 QTLs were common to S840 and S844. We found that when the genotype at the detected QTLs was homozygous for ‘SekihoS8’, the plant showed early flowering (Figure S6).

**Figure 3:**
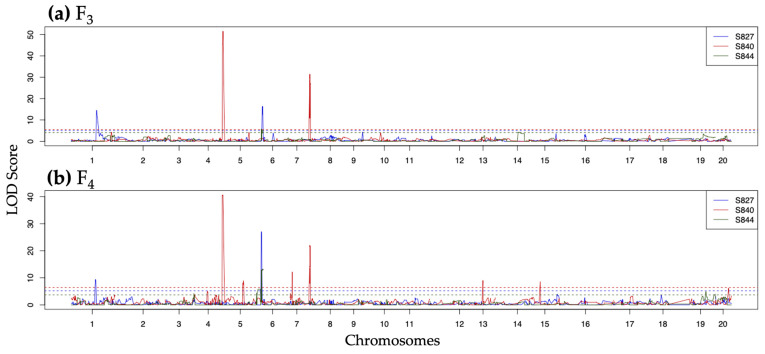
Quantitative trait loci detected for perillaldehyde in S827 (blue), S840 (red), and S844 (green): (**a**) QTLs detected in the F_3_ population; (**b**) QTLs detected in the F_4_ population. Dashed lines represent the logarithm of odds (LOD) threshold estimated by 10,000 permutations for each population.

Among the QTLs detected in PA, 16 were common to S827 and S844 on chr5. Twenty-eight QTLs were detected in S840 on chr5 and chr7, which were common to the F_3_ and F_4_ generations and were at different positions from the QTLs detected in the other two populations (Figure 3). For QTLs commonly detected in S827 and S844, plants with genotypes homozygous for ‘SekihoS8’ had lower PA content (Figure S7a). In contrast, for QTLs detected only in S840, plants with genotypes homozygous for ‘SekihoS8’ had high PA contents, whereas those with genotypes homozygous for ‘st40’ had a PA content close to zero (Figure S7b).

### 3.2. Genomic Heritability

The genomic heritability of all the traits mentioned in Section 2.2, estimated using each population and the three populations combined, is shown in Table 1 and Table 2. Genomic heritability varied substantially among traits and populations. Among all traits, RA had the lowest genomic heritability, with values ranging from 0.151 to 0.484. In contrast, PA, ANT, yield, and flowering date showed considerably higher genomic heritability. Among the three populations, the genomic heritability estimated using S844 was consistently the lowest for almost all traits except yield. When genomic heritability was estimated using the three populations combined, it was always greater than the average genomic heritability estimated using each population singularly. For the flowering date, the genomic heritability estimated using the three populations combined was 0.879, which was the highest among all estimates made using each population.

### 3.3. Genetic Correlation

**Figure 4:**
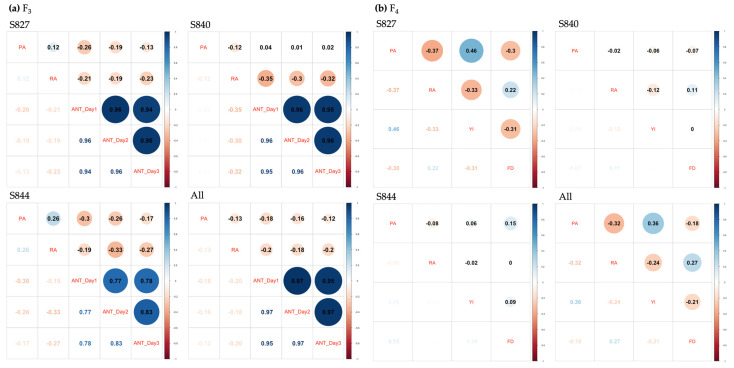
Genetic correlation among traits using each population and the three populations combined: (**a**) Genetic correlations using the F_3_ population; (**b**) genetic correlations using the F_4_ population. PA: perillaldehyde, RA: rosmarinic acid, ANT_Day n: n^th^ measurement of anthocyanin, YI: yield, FD: flowering date.

The multi-trait GBLUP model was used to estimate the genetic correlations among all traits. As shown in Figure 4, variations were observed in the positivity, negativity, and magnitude of genetic correlations among different generations and populations. However, for ANT, a strong positive correlation was observed across the three measurements in all populations. For genetic correlations estimated using three populations combined, two prime medicinal compounds, PA and RA, showed weak negative correlations with ANT (−0.2 to −0.12), while PA and yield showed a moderate positive correlation. The genetic correlation between these two traits became more negative from the F_3_ to the F_4_ generation, except in S840. When genetic correlations were estimated for each population, S840 had the lowest absolute values for almost all traits.

### 3.4. Accuracy of Genomic Prediction

**Figure 5:**
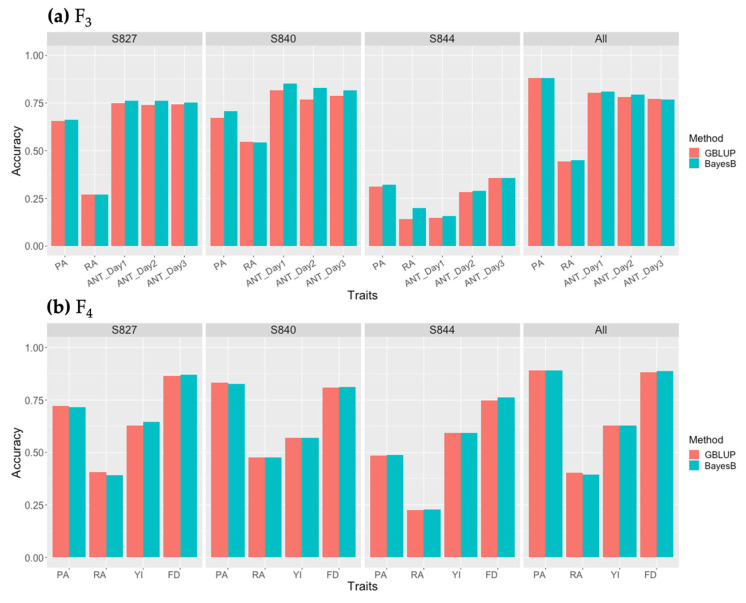
Prediction accuracy of single-trait genomic prediction (GP) by the GBLUP (red) and BayesB (cyan) models using each population and the three populations combined: (**a**) prediction accuracy using the F_3_ population; (**b**) prediction accuracy using the F_4_ population. PA: perillaldehyde, RA: rosmarinic acid, ANT_Day n: n^th^ measurement of anthocyanin, YI: yield, FD: flowering date.

GP models were constructed for each F_3_ and F_4_ generation using each population and the three populations combined. The prediction accuracy of the two single-trait models (GBLUP and Bayes B) is shown in Figure 5. Traits and populations with low genomic heritability tended to have a lower GP accuracy. When the models were constructed using each population, regardless of the GP model, S844 had the lowest accuracy for almost all traits, while S827 and S840 had the highest accuracy for yield and flowering dates and PA, RA, and ANT, respectively. The models constructed using the three populations combined showed improved accuracy compared with the models constructed using each population for PA and flowering date. When comparing the accuracies of the GBLUP and BayesB models, the BayesB model had better prediction accuracies for most traits, with values ranging from 0.0466% to 41.7%, higher than those of GBLUP. However, the GBLUP model was superior for some traits and populations, particularly RA, with a 0.0547–4.25% higher accuracy than the BayesB model. Furthermore, the same GP model showed higher prediction accuracy using the F_4_ population than when using the F_3_ population. When the multi-trait models (GBLUP and BayesCπ) were used, the prediction accuracies obtained were similar to those obtained using the single-trait model (Figure S8).

## 4. Discussion

In this study, we conducted QTL and GP analyses for the major medicinal compounds and other agronomic traits of *P. frutescens* to reveal the genetic mechanisms underlying these traits and to evaluate the efficiency of GS and MAS. Genetic correlations, heritability, and GP accuracy revealed that each population had varying characteristics; however, reasonable values were obtained when the three populations were used together. The results of the QTL analysis for each trait showed that traits such as PA, ANT, and flowering date had significant QTLs with high LOD scores; however, no significant QTLs were detected for RA. One possible reason for this could be that no significant differences in RA content among the four cross-parents were observed, as shown in Table S1, and all parents already had genes that had a large effect on RA. These results revealed the genetic characteristics of each trait. Examination of the association between marker genotypes at the QTL and phenotypes of ANT revealed that plants with the ‘SekihoS8’ homozygous genotype had a significantly higher ANT content. ‘SekihoS8’ is a red perilla variety, which has been reported to accumulate more ANT in the leaves [2,3]. In contrast, the green perilla line, another cross-parent, has been reported to accumulate minimal amounts of ANT in its leaves. Therefore, the detected QTLs were considered to be important gene loci associated with the ANT metabolic pathway. Zhang et al. performed a genome-wide association study for ANT and detected a strong signal on chr8, which was assumed to be the MYB transcription factor regulating ANT biosynthesis in vegetative plant tissues [30]. The two QTLs for ANT detected in our study were also located on chr8 and had extremely high LOD scores, consistent with those reported by Zhang et al. [30]. QTLs for yield were identical to those for ANT, but yield tended to be lower when the genotype at these QTLs was homozygous for the ‘SekihoS8’ allele. The QTLs for ANT had a contradictory effect on yield; however, simultaneous enhancement of both traits during breeding was not difficult, as the yield was also substantially affected by other markers. Therefore, individuals with high ANT content can be selected by MAS using these markers, and an improvement in yield can be achieved by combining the effects of other markers with GS. For PA, the QTLs detected in S840 differed from those detected in S827 and S844. When the marker genotypes of the QTLs detected only in S840 were homozygous for st40, PA content was nearly zero. PA is an essential oil that produces a strong odor. During cultivation, st40 differed considerably from st27 and st44. These results suggest that plant PA production is controlled by QTLs located on chr5 and chr7 of S840. Furthermore, among the perilla plants that produce PA, QTLs that determine the amount of accumulated PA may be located at different positions on chr5, which were detected in S827 and S844. Among the detected QTLs, 1 QTL was common to both S827 and S844, and 10 QTLs were common to both S840 and S844. Kang et al. performed QTL analysis on traits related to flowering time and detected six QTLs that were identified as perilla orthologs regulating flowering time [31]. However, the linkage groups in their study did not converge to 20 (the number of chromosomes in perilla). Therefore, we cannot confirm whether this is consistent with our study.

According to the genomic heritability and GP estimation, all traits except RA had considerably high genomic heritability, indicating that improvement through GS would be effective for these traits. Even for RA, which had the lowest genomic heritability among all traits, the genomic heritability was approximately 0.4 in S827 and S840. Since we evaluated the genomic heritability of each trait based only on additive genetic effects, selection accuracy could be further improved by considering non-additive genetic effects, such as dominance and epistatic effects, in the GP model, even for traits with low genomic heritability, such as RA [32].

Regarding the comparison of the prediction accuracy between different GP models, the BayesB and GBLUP models differed depending on the traits. This is because the fitness of either model depends on whether each trait is controlled by a small number of QTLs with large effects or by an accumulation of polygenes with small effects. For most traits, the Bayes B model was more accurate than the GBLUP model; however, for RA, the GBLUP model was 4.2% more accurate than the Bayes B model. This indicates that RA is regulated by numerous genes. In contrast, PA, ANT, and flowering date were significantly affected by only a few genes. Such differences in the genetic mechanisms of the traits were also evident in the QTL analysis.

The accuracy of genomic selection depends not only on genomic heritability but also on factors such as the strength of linkage disequilibrium (LD), number of QTLs, and population size [33]. For traits with low heritability, marker density may affect the accuracy of GP, with some studies showing that the accuracy improved when LD between adjacent markers was >0.2 [34]. Considering the relationship between marker density and GP prediction accuracy, of the three populations, S840 showed the highest prediction accuracy for most traits, probably because 1432 SNPs were used for prediction. This was approximately 2.5 times higher than that of the 579 SNPs used in S844, which had the lowest prediction accuracy among the three populations. Therefore, increasing the population size or number of markers may further improve the prediction accuracy for S844 and traits such as RA.

Furthermore, evaluation of the multi-trait GP model showed that the prediction accuracy was similar to that of the single-trait GP model. One of the reasons for this may be that the genetic correlation among the traits estimated in Section 3.3 was not strong. Another notable result was that the genetic correlations among the traits differed for each population. During breeding, the absence of strong negative correlations between target traits is highly desirable. However, the results revealed that some populations showed slightly negative genetic correlations for some traits. These negative genetic correlations can be reduced by simultaneously using multiple populations with different genetic backgrounds during breeding.

Finally, we examined the possibility of the genomic breeding of red perilla as a medicinal plant and discussed the different genetic mechanisms underlying the regulation of medicinal and other agronomic traits. Therefore, it is important to use various GP models and selection methods suitable for the genetic characteristics of each trait. For traits such as PA and ANT, where significant QTLs have been detected, selection can be performed using MAS. Even for traits such as RA, where no highly effective QTL is present in a breeding population to be used, selection can be made based on GP. Even for traits that are controlled by a QTL with a large effect, further improvement can be expected by using GS to accumulate genes with a small effect. Because the populations used in this study had only four cross parents and because of the strong linkage disequilibrium at the chromosome level in the F_3_ and F_4_ populations, the number of markers and population size on a small scale was sufficient for prediction accuracy. To breed medicinal plants, it is more realistic to create relatively small breeding populations for the application of MAS and GS. However, if one wants to make the GP model more general and more accurate for application to other populations, the model should be built using a large number of populations containing more genetic diversity [35]. Moreover, the results of this study indicate that the simultaneous use of multiple populations with different characteristics, such as different genetic correlations among target traits and different QTL positions, for breeding can result in multiple trait improvements when the size of each population is sufficient to build the prediction model. However, there are limitations in applying the GP models used in this study to multiple populations with varying genetic backgrounds in a general case. In the GP models using the three populations combined in this study, the effect of each population was included in the model as differences in the population mean (intercept), which may not be sufficient to account for differences in genetic background between populations. A possible way to model the genetic background between populations is to use a GP model that assumes that different populations have different genetic variances for the same QTL [36,37].

## 5. Conclusions

In this study, based on the results of the QTL analysis and the prediction accuracy of GP, the use of GS and MAS instead of conventional phenotypic selection proved to be effective in red perilla breeding. Because each trait has a different genetic mechanism, MAS should be used for traits in which large QTLs are detected, whereas GS should be used for traits controlled by polygenes. In addition, using multiple populations with different genetic backgrounds for breeding at the same time may lead to the simultaneous improvement of multiple traits. However, further research is required for its generalization. Our research contributes to the study of the genetic mechanisms underlying the production of medicinal compounds in perilla, as well as to the acceleration of medicinal plant breeding using MAS and GS.

## Figures and Tables

**Table 1:** Estimated genomic heritability in F_3_ generation using each population and three populations combined.

Population	PA	RA		ANT	
			Day 1	Day 2	Day 3
S827	0.626	0.227	0.718	0.707	0.721
S840	0.505	0.484	0.803	0.726	0.760
S844	0.190	0.186	0.114	0.241	0.355
All	0.548	0.401	0.661	0.644	0.639

PA, perillaldehyde; RA, rosmarinic acid; ANT, day n; n^th^ measurement of anthocyanins.

**Table 2:** Estimated genomic heritability in F_4_ generation using each population and three populations combined.

Population	PA	RA	YI	FD
S827	0.766	0.394	0.634	0.869
S840	0.796	0.389	0.605	0.848
S844	0.449	0.151	0.715	0.691
All	0.735	0.350	0.702	0.879

PA, perillaldehyde; RA, rosmarinic acid; YI, yield; FD, flowering date.

## Data Availability

All other relevant data are available upon reasonable request.

## Supplementary Materials

The following supporting information can be downloaded from https://www.mdpi.com/article/10.3390/genes14122137/s1: Table S1: Phenotype data of four cross-parents collected in 2022. Figure S1. Common genetic maps constructed using 20 chromosomes defined from three populations. Figure S2: QTLs detected for rosmarinic acid in S827, S840, and S844. Figure S3: QTLs detected for anthocyanins in S827, S840, and S844. Figure S4: QTLs detected for yield in S827, S840, and S844. Figure S5: QTLs detected for flowering dates in S827, S840, and S844. Figure S6: Genotypes of QTL detected on chr19 for flowering date. Figure S7: Genotypes of QTLs detected for perillaldehyde. Figure S8: Prediction accuracy of multi-trait GP by the GBLUP and BayesCπ models using each population and the three populations combined.
