# Supplementary material for: Assessing the Potential for Genome-Assisted Breeding in Red Perilla Using Quantitative Trait Locus Analysis and Genomic Prediction
Source: Genes (Basel). 2023 Nov 27;14(12):2137. doi: 10.3390/genes14122137 (PMC10742415; doi:10.3390/genes14122137)
Supplement: Supplementary file 1 [file genes-14-02137-s001.zip › genes-2672366-supplementary.pdf]

Table S1. Phenotype data of four cross-parents collected in 2022.

Parents	PA (mg/g DW)	RA (mg/g DW)	ANT	YI (g DW)	FD
SekihoS8	5.37	23.87	Red	46.47	140
st27	6.36	17.64	Green	81.13	130
st40	0.34	18.51	Green	83.67	144
st44	4.74	25.12	Green	90.93	146

PA, perillaldehyde; RA, rosmarinic acid; ANT, anthocyanin; YI, yield; FD, flowering date.

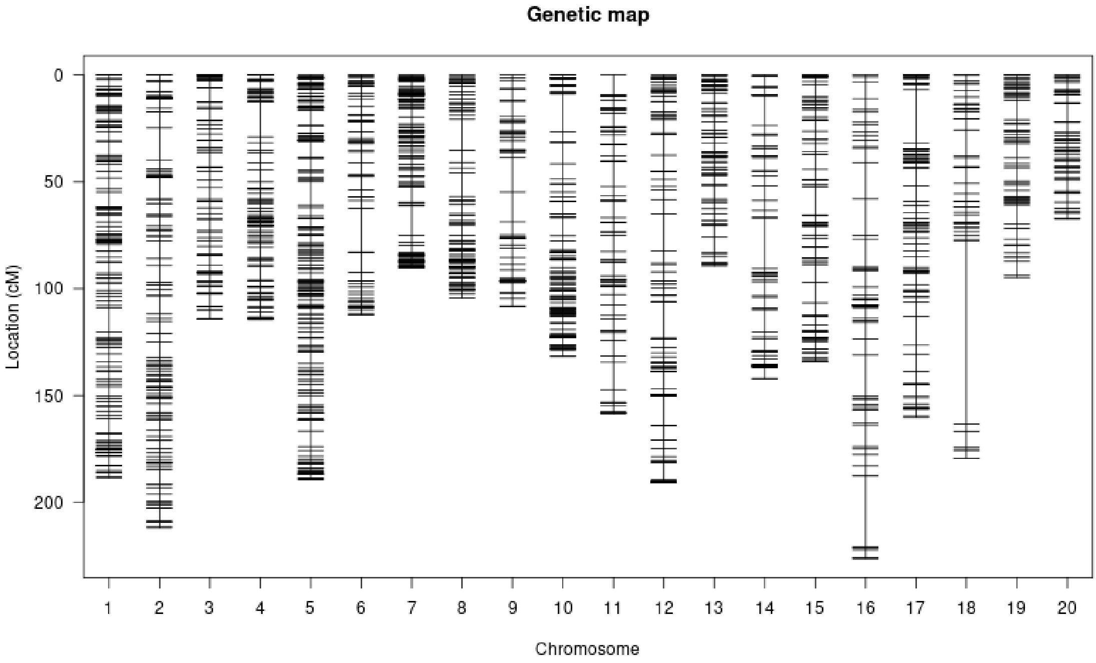

Figure S1. Common genetic maps constructed using 20 chromosomes defined from three populations.

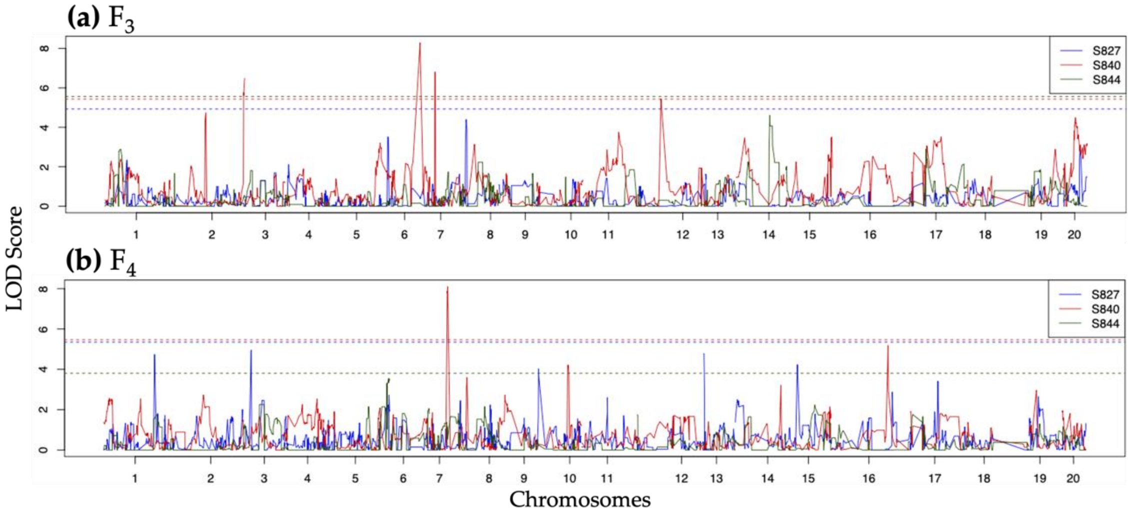

Figure S2. QTLs detected for rosmarinic acid in S827 (blue), S840 (red), and S844 (green). **(a)** QTLs detected in the F_3 population; **(b)** QTLs detected. in the F_4 population. Dashed lines represent the LOD threshold estimated by 10,000 permutations for each population.

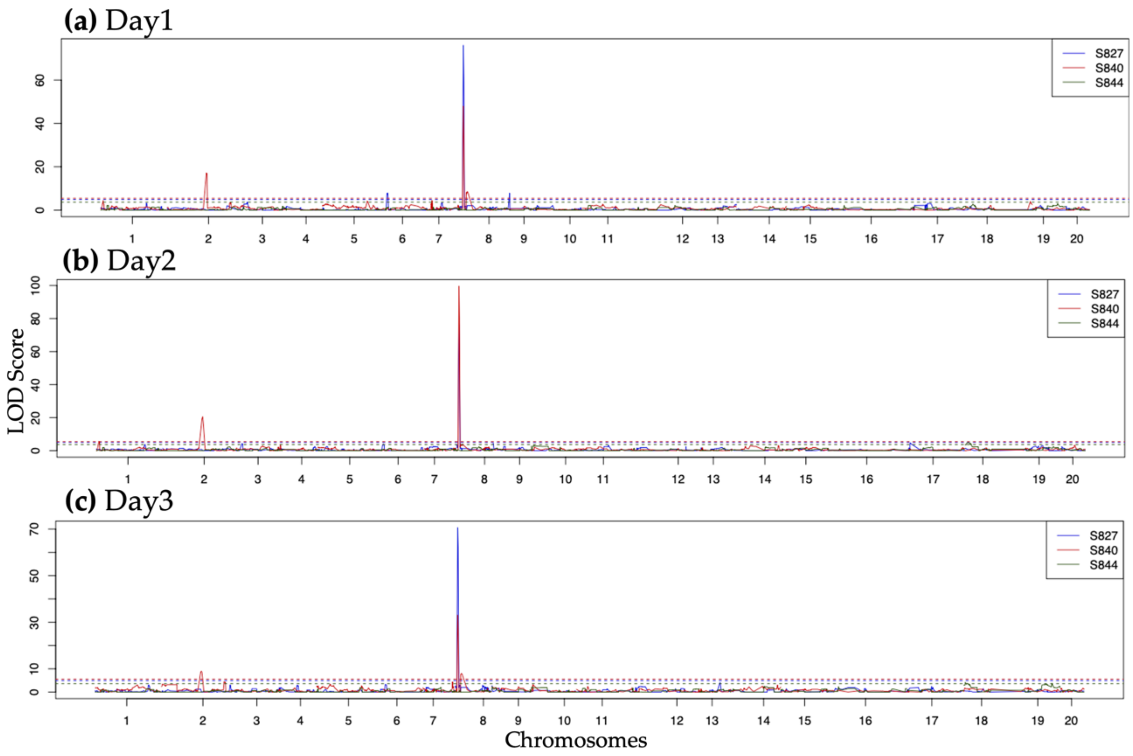

Figure S3. QTLs detected for anthocyanin in S827 (blue), S840 (red), and S844 (green). **(a)** QTLs detected in the 1st measurement of anthocyanin; **(b)** QTLs detected in the 2nd measurement of anthocyanin; **(c)** QTLs detected in the 3rd measurement of anthocyanin. Dashed lines represent the LOD threshold estimated by 10,000 permutations for each population.

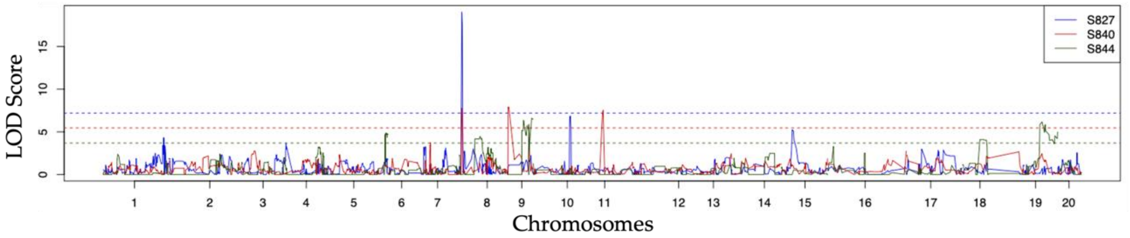

Figure S4. QTLs detected for yield in S827 (blue), S840 (red), and S844 (green). Dashed lines represent the LOD threshold estimated by 10,000 permutations for each population.

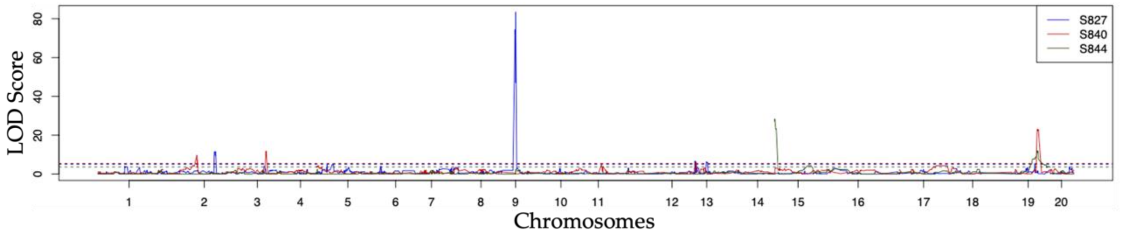

Figure S5. QTLs detected for flowering date in S827 (blue), S840 (red), and S844 (green). Dashed lines represent the LOD threshold estimated by 10,000 permutations for each population.

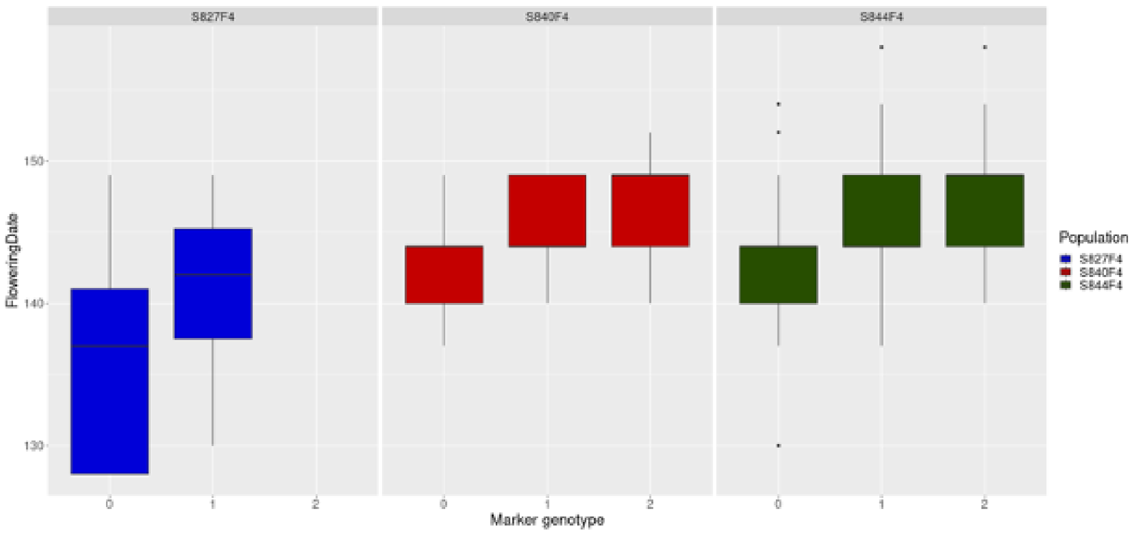

Figure S6. Genotypes of QTL detected on chr19 for flowering date. Genotype scores 0, 1, and 2 represent homozygous for 'SekihoS8', heterozygous, and homozygous for the other cross parents, respectively. Blue: S827; Red: S840; Green: S844.

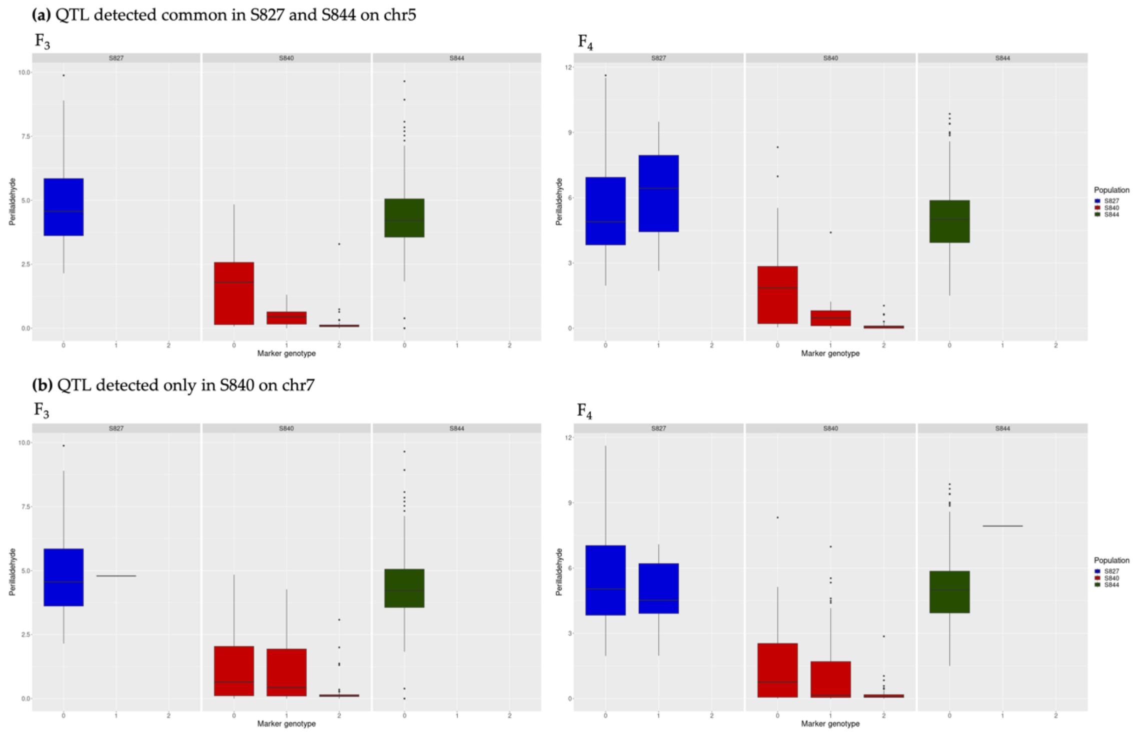

Figure S7. Genotypes of QTLs detected for perillaldehyde. Genotype scores 0, 1, and 2 represent homozygous for 'SekihoS8', heterozygous, and homozygous for the other cross parents, respectively. The panel on the left side is for F_3 generation and the panel on the right side is for F_4 generation. Blue: S827; Red: S840; Green: S844. (**a**) The genotypes at the QTL detected in both S827 and S844 on chr5; (**b**) The genotypes at the QTL detected only in S840 on chr7.

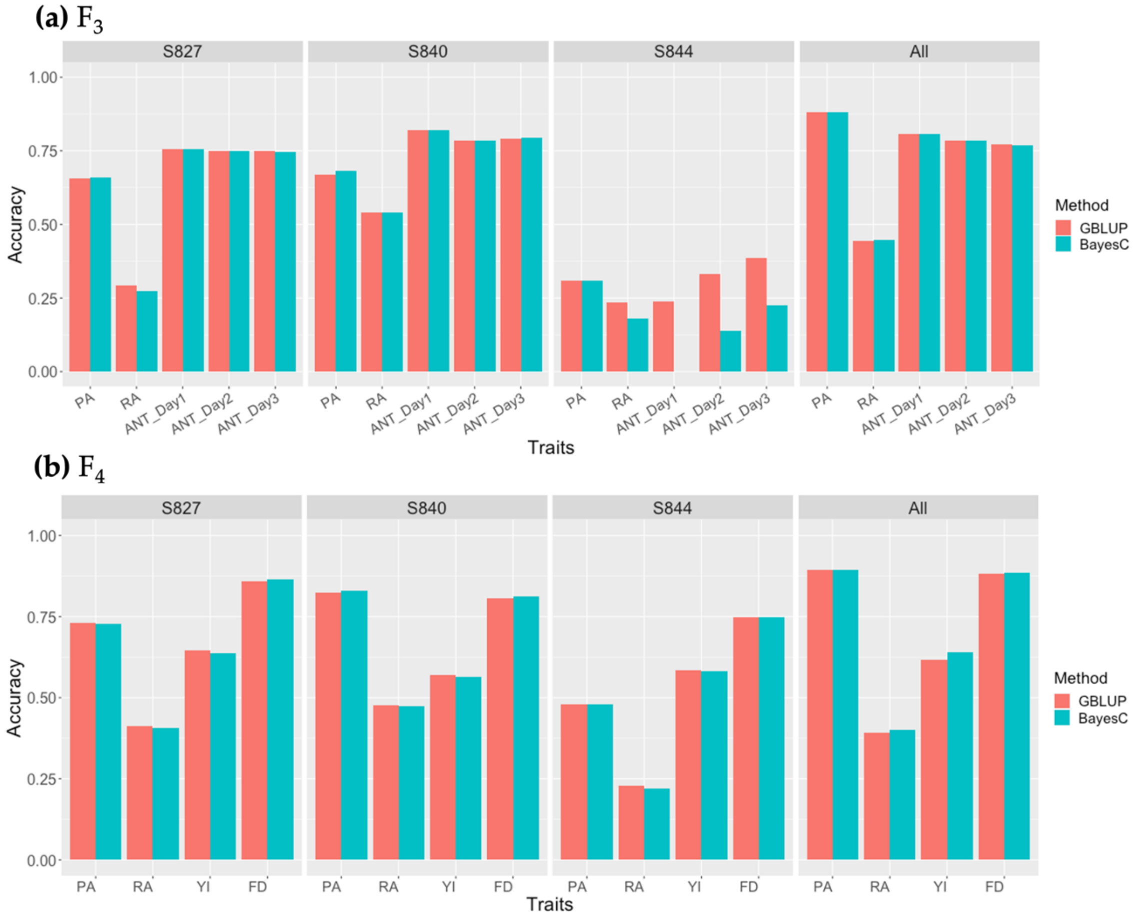

Figure S8. Prediction accuracy of multi-trait GP by the GBLUP (red) and BayesCπ (cyan) models using each population and the three populations combined. **(a)** Prediction accuracy using the F_3 population; **(b)** Prediction accuracy using the F_4 population. PA: perillaldehyde, RA: rosmarinic acid, ANT_Dayn: n^{th} measurement of anthocyanin, YI: yield, FD: flowering date.
